# Implementing a Standardized Language Evaluation in the Acute Phases of Aphasia: Linking Evidence-Based Practice and Practice-Based Evidence

**DOI:** 10.3389/fneur.2020.00412

**Published:** 2020-06-01

**Authors:** Megan E. Schliep, Laura Kasparian, Olga Kaminski, Carla Tierney-Hendricks, Esther Ayuk, Lynne Brady Wagner, Semra Koymen, Sofia Vallila-Rohter

**Affiliations:** ^1^MGH Institute of Health Professions, Boston, MA, United States; ^2^Speech-Language Pathology Department, Spaulding Rehabilitation Hospital, Boston, MA, United States; ^3^Speech-Language and Swallow Department, Brigham and Women's Hospital, Boston, MA, United States

**Keywords:** implementation science, aphasia, standardized assessment, acute care, rehabilitation, stroke recovery

## Abstract

The research to practice gap is a significant problem across all disciplines of healthcare. A major challenge associated with the adoption of evidence into routine clinical care is the disconnect between findings that are identified in a controlled research setting, and the needs and challenges of a real-world clinical practice setting. Implementation Science, which is the study of methods to promote research into clinical practice, provides frameworks to promote the translation of findings into practice. To begin to bridge the research-practice gap in assessing recovery in individuals with aphasia in the acute phases of recovery following stroke, clinicians in an acute care hospital and an inpatient rehabilitation hospital followed an implementation science framework to select and implement a standardized language assessment to evaluate early changes in language performance across multiple timepoints. Using a secure online database to track patient data and language metrics, clinically-accessible information was examined to identify predictors of recovery in the acute phases of stroke. We report on the feasibility of implementing such standardized assessments into routine clinical care via measures of adherence. We also report on initial analyses of the data within the database that provide insights into the opportunities to track change. This initiative highlights the feasibility of collecting clinical data using a standardized assessment measure across acute and inpatient rehabilitation care settings. Practice-based evidence may inform future research by contributing pilot data and systematic observations that may lead to the development of empirical studies, which can then feed back into clinical practice.

## Introduction

Two million people in the United States are living with aphasia—an impairment in language comprehension and production. Speech-language pathologists play a central role in the assessment and diagnosis of individuals with language deficits following stroke, and current clinical practice for the assessment of language skills following stroke is variable across and within clinical practice settings (1). Lack of consistency places limitations on the understanding of early stroke recovery, and limits care continuity between settings and clinicians. Further, there is a disconnect between what occurs within clinical practice and advancements being made in research to inform recovery predictions.

The research to practice gap, defined as the discrepancy between evidenced-based interventions and what takes place in practice, has been well-documented (2–6). Studies have suggested that it takes 17 years for 14% of healthcare research to be adopted into routine clinical practice (7). This slow translation of research to the clinic is one of the disconnects that confines healthcare and clinicians' abilities to optimize care for patients. The limited uptake of research has been attributed to a variety of factors, including the level of relevance of research findings to practice, organizational constraints that impact the adoption of findings into practice, and the degree of benefit to the target population to sustain the practice (4, 6, 8).

Evidence-based medicine calls for the integration of the best available evidence from systematic research in the care and clinical decision-making process for individual patients (9). The goal of EBP involves the integration of (1) external scientific evidence, (2) clinical expertise, and (3) client, patient, and caregiver values and perspectives (9–11). A major challenge associated with the adoption of EBP, however, is the disconnect between the findings identified in a controlled lab setting and those that are ultimately implemented in a real-world clinical practice setting (3, 12–16). The scientific pipeline has generally prioritized scientific control for internal validity; while categorically important, the focus on internal validity may come at the expense of external validity, or generalizability across setting and time (3). By bringing research closer to the actual practice setting and creating practice-based evidence, results may be more relevant, tailored, and actionable to patients and clinicians (3, 17–19).

Important for any attempts to bring research close to the practice setting is Implementation Science. Implementation Science is the study of methods that promote systematic uptake of research into routine clinical practice (8, 20–22), offering frameworks and structure to help guide successful implementation [e.g., (22–24)]. Additionally, practice-based evidence, the concept that clinicians can structure practice and measure outcomes in the real-world care setting, offers an opportunity to inform research needs and speed the research to practice transfer (3, 17–20, 25).

Prior work has demonstrated that, with the guidance of implementation science frameworks, a standardized process for the evaluation of language was feasible in acute care and improved diagnosis and reporting of aphasia [see (26)]. The current manuscript describes a follow-up study that reports on the long-term adherence to the implemented measure, and on the extension to an inpatient rehabilitation facility. Clinicians in acute care and inpatient rehabilitation hospitals, both within the same healthcare network, have been working together with the long-term goal of populating a database with consistent measures of language performance across the early stages of aphasia diagnosis to begin to inform early language recovery patterns. Stroke-related information, including lesion size and lesion location, have been identified as key factors in predicting language outcomes, and initial aphasia severity has been acknowledged as the most robust factor in predicting language recovery (27–33). The extent to which clinicians make recovery predictions, however, and share these with their patients is limited. One of the reasons research knowledge about recovery has not translated to the clinic is that current predictive information is not fine-grained enough to capture clinically-observable skills at the individual level, or they require high-level analyses that are more consistent with the research setting. Standardizing clinical practice to gather data may help shed light on the types of data that are feasible to capture clinically and could be informative to outcomes.

Thus, to begin to bridge the research-practice gap in predicting language abilities in individuals with aphasia in the acute phases of recovery following stroke, this manuscript reports on (1) the feasibility of adhering to a standardized language assessment protocol in acute care over a 2-year period, (2) the iterative implementation process utilized in an inpatient rehabilitation care facility following an implementation science framework, and (3) a pilot evaluation of data collected through standardized assessments to begin to evaluate predictive models of language recovery after stroke.

### Part I—The Feasibility of Adhering to a Standardized Language Assessment Protocol in Acute Care

**Figure 1:**
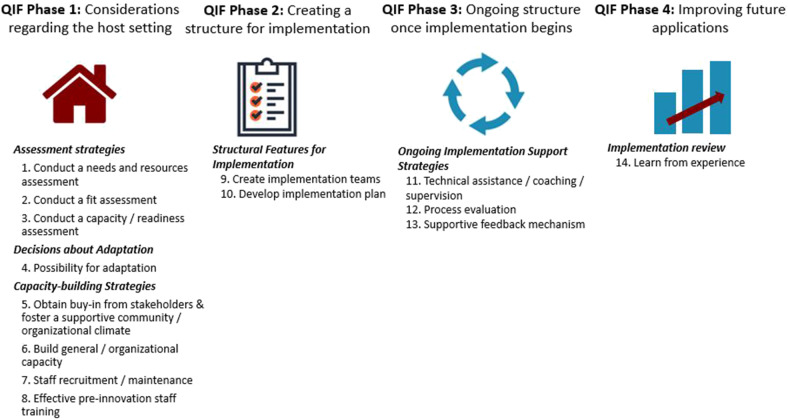
Fourteen critical steps of quality implementation according to the Quality Implementation Framework (QIF) established by Meyers et al. (22).

Between October of 2016 and June of 2017, an iterative process of implementation was carried out at Brigham and Women's Hospital (BWH) to standardize the process of language evaluation. BWH is a 777-bed acute-care teaching hospital of Harvard Medical School within the Partners HealthCare Network. The hospital transitioned from paper medical records to electronic medical records in 2015, which created an opportunity for clinicians to assess clinical practices and consider how to most effectively integrate clinical expertise within the new documentation structure. The goal of this implementation project was to identify a clinical process to improve the evaluation and diagnosis of aphasia within the constraints of the acute care setting and to maximize efficiency and clarity of information within the electronic medical record. In brief review [see (26) for full report], a team of researchers and clinicians formed an implementation team and carried out the implementation process using the fourteen-step, four phase, Quality Implementation Framework (QIF) proposed by Meyers et al. [(22), see Figure 1].

QIF Phase 1 (considerations of the host setting), readiness for change was facilitated by the transition to the electronic record. During QIF Phase 2 (creating a structure for implementation), a literature review was performed by the implementation team to select an assessment that was feasible to administer in acute care that addressed implementation goals of improved diagnosis [the Western Aphasia Battery-Bedside Version (WAB-Bedside)], and software was selected to support data collection and entry into the medical record [REDCap, a secure online database supported by Partners HealthCare (34)]. In QIF Phase 3 (maintenance of the structure once implementation begins), a screening tool was developed to assess patient ability to participate in assessment given that certain patients seen in acute care were not sufficiently alert and oriented to attempt purposeful responses [see (26) for additional details regarding the screener], training sessions were held to educate staff about the measure, and surveys were collected to gather data about evolving practice patterns and needs. QIF Phase 4 (improving future applications) involved evaluation of the implementation to improve future practice. Evaluation of the implementation, carried out through medical record review of 50 (25 post-implementation and 25 pre-implementation) records demonstrated improved consistency of reporting on language domains of repetition ability, naming ability, yes-no question response accuracy, and awareness of errors, as well as a significant increase in the reporting of specific aphasia diagnosis (26). In addition to quantifiable improvements, the team felt that administering a standardized measure helped improve handoff communication and streamlined practice. Therefore, in July of 2017 training sessions were held to expand the standardized measure to the entire BWH clinical team. In this follow-up study, we aimed to determine adherence to the standardized protocol over the two-year period since expansion to the clinical team.

## Methods

Based on processes established via the implementation referenced above, since 2017, when a consult was placed requesting a language evaluation at BWH, patients were screened to determine if they were sufficiently alert to complete the standardized assessment. If passed, participants were given the spoken and auditory comprehension portions of the WAB-Bedside (35), and data were entered directly into an online database supported by REDCap. The onboarding of new staff involved training on the administration of the standardized assessment and on the data entry process in REDCap by supervisors and senior staff. Once new clinicians were ready to administer the measure in their clinical practice, they were observed by a senior clinician who provided feedback on administration. Clinicians were accompanied by a senior clinical team member until they were judged to adhere to the standardized protocol. Standardized evaluation procedures were reinforced quarterly through staff meetings.

Retrospective medical record review was conducted to evaluate adherence to the standardized evaluation process in acute care. This retrospective medical record review was approved by the Institutional Review Board of Partners HealthCare. Partners HealthCare has a Research Patient Data Registry (RPDR), that allows data to be queried based on the International Classification of Diseases (ICD-10) diagnosis codes. Using the RPDR, we identified patients older than 18 who were admitted to BWH from July 2017 to August 2019 with diagnosis codes that contained the search terms: speech and language deficits (following cerebrovascular disease, cerebral infarction, hemorrhage etc.), aphasia, cognitive deficits, cognitive impairment, cognitive functions, and brain neoplasm (see Data Sheet 1 for a full list of query items). Billing data from these queries were searched for Current Procedural Terminology (CPT) billing codes 92523 (Evaluation of speech sound production; with evaluation of language comprehension and expression and 96105 (Assessment of aphasia and cognitive performance testing), the two billing codes used at BWH for language evaluations. In this manner, medical record numbers for patients admitted to BWH who received language evaluations were identified. Duplicate entries were removed and billing data was compared to language evaluation data in the REDCap database to determine the percentage of language evaluations that were performed using the standardized process over the 2-year period.

## Results

The RPDR data pull resulted in 371 entries corresponding to patients who were billed for receiving a language evaluation in the period from June 2017 until August 2019. These patients represented primary diagnoses that included cerebral infarction, non-traumatic hemorrhage, and malignant neoplasm. An examination of adherence demonstrates that of the 371 entries, 260 individuals (70.1%) received the standardized assessment protocol.

### Part II—The Iterative Implementation Process Utilized in an Inpatient Rehabilitation Care Facility Following an Implementation Science Framework

In early 2016, clinicians at Spaulding Rehabilitation Hospital (SRH), an acute rehabilitation hospital within the Partners HealthCare Network, recognized the need for standardization and began trialing a standardized language assessment tool with all patients admitted to the Stroke Rehabilitation Program, where all admitted patients carry a diagnosis of stroke. The standardization process followed an informal procedure until 2017, when a collaboration was formed with Brigham and Women's Hospital.

## Methods

**Figure 2:**
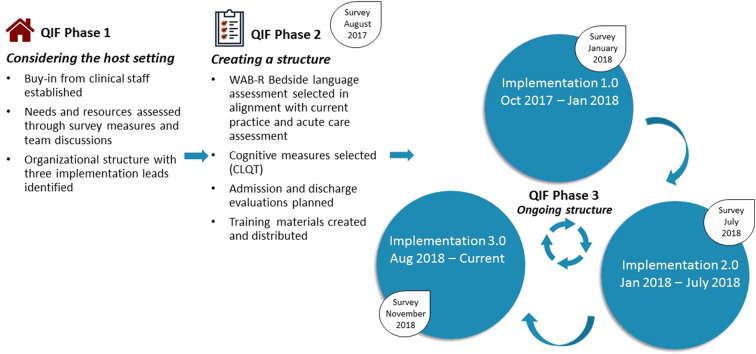
Summary of QIF Process at Spaulding Rehabilitation Hospital. **Implementation 1.0** included the WAB Bedside, as well as four subtests from the CLQT (Clock Drawing, Symbol Cancellation, Design Memory, Design Generation). Assessment of reading and writing skills was formalized to improve consistency of administration. The assessment measure was administered to all patients with CVA admitted to the Stroke Program. **Implementation 2.0** included the addition of a screening tool, administration of the full WAB (rather than WAB Bedside) upon admission for individuals with L MCA stroke, and re-evaluation via the WAB Bedside at 10–14 days post admission. **Implementation 3.0** included training and expansion for assessment administration by all clinicians, including full-time employees and per diem weekend staff, as well as expansion to CVA admissions hospital-wide (rather than just those admitted to the Stroke Program).

In June of 2017, based on prior work, teams worked together to initiate an iterative implementation based on the four phases of the quality improvement framework (QIF) for implementation proposed by [Meyers et al. (22)] with the goal of aligning procedures and resources across the two facilities. Three phases of QIF have been implemented, with key considerations and/or changes identified in Figure 2. Survey measures were administered to clinical staff at each phase of implementation to gather feedback and evaluate for potential improvements.

### QIF Phase 1: Initial Considerations Regarding the Host Setting

Initial considerations of the host setting demonstrated that buy-in from stakeholders was already established, as the SRH clinical team had recognized the need for standardization 1 year prior. SRH was using the WAB-bedside assessment, which aligned with the measure implemented at BWH, making alignment of measures readily feasible. An organizational structure was implemented, with three members of the clinical team identified as implementation leads. A survey was distributed to clinicians to identify aspects of current practice that were effective and those that might be improved upon (see Data Sheet 2). Implementation leads held meetings with BWH researcher-clinicians to gather insights about the process at BWH and establish a plan for SRH.

### QIF Phase 2: Creating a Structure for Implementation

In addition to the WAB-bedside language evaluation, data on cognitive measures was important for SRH clinicians to gather, therefore, four subtests of the Cognitive Linguistic Quick Test [CLQT (36)] were added to the standardized process. In addition, it was determined that re-evaluation prior to discharge would be meaningful to evaluate change, and re-administration of the WAB-bedside and CLQT subtests was targeted to occur within 48 h of planned discharge. While at BWH, WAB-bedside data were entered directly into a REDCap database, the CLQT is not as seamlessly administered on a computer and SRH clinicians felt paper administration was more conducive to the rehabilitation environment, therefore a decision was made to use traditional paper and pencil formats for both assessments. It was decided that the standardized implementation would first be carried out only by full time clinicians within the Stroke Program of SRH. Staff training occurred through meetings and printed materials distributed throughout the Stroke Program. This plan was put into place in October of 2017 and maintained until January 2018 and was referred to as Implementation 1.0.

### QIF Phase 3: Ongoing Structure Once Implementation Begins

At the end of implementation 1.0, a survey was distributed to full-time clinical staff. Survey responses and observations from implementation leads revealed that clinicians felt that the WAB-bedside was not sufficient, in many cases, to evaluate language abilities for individuals having experienced left middle cerebral artery (MCA) or anterior cerebral artery (ACA) cerebrovascular accidents, who are those who most consistently present with aphasia. A more comprehensive evaluation was requested. In addition, re-administering measures was difficult to do 48-h prior to discharge in the setting of shifting discharge plans and caseloads. In response to these observations, the standardized process was modified to (1) include different language assessments for patients experiencing left hemisphere strokes (Full WAB-R) vs. those affecting the right hemisphere and/or cerebellum (WAB-Bedside) (see Supplementary Figure 1), and (2) schedule re-testing to take place 10–14 days after initial assessment so that calendar alerts could be programmed and re-testing scheduled. This structure (Implementation 2.0) was carried out until July 2018, when another survey was administered. Survey data and implementation guided observation led to Implementation 3.0 characterized by the creation of templates to guide write-ups of evaluations and expansion of the measure to include per diem staff for improved consistency. Educational materials about the standardized process were distributed to all staff and full time clinicians were identified as point-people for per diem clinicians. To establish a process for evaluating data, research staff also joined the project, and, on a weekly basis, a research assistant at the MGH Institute of Health Professions pulled standardized language evaluation data from the Spaulding electronic medical record into the REDCap database. Implementation 3.0 was carried out on the Stroke Program from August 2018 until December 2018. This period is referred to as Implementation 3.0-Stroke Program. In January 2019, educational meetings were held and the standardized process was expanded to include clinicians on other services within SRH also involved in language evaluations. The period from January 2019 to August 2019 is referred to as Implementation 3.0-Hospital.

### Evaluation of Adherence

Adherence to the implementation measure was evaluated over Implementation 3.0-Stroke Program and Implementation 3.0-Hospital. To do so, Spaulding Rehabilitation Hospital admission data were retrieved for all patients admitted with diagnosis classifications of stroke rehabilitation, physical medicine and rehabilitation (PMR) stroke, Acute neurology stroke, PMR neurology, neurology and brain injury. For the period from August 2018 to December 2018, data were filtered to only consider patients admitted to the Stroke Program. From January 2018 to August 2019 all stroke admissions data were included (Implementation 3.0-Hospital). Admissions data were then compared with REDCap data to determine whether patients received the standardized protocol or another assessment procedure.

## Results

From August 2018 to December 2018, there were a total of 169 admissions to the Stroke Program comprising 79 with L MCA/ACA strokes and 90 with other stroke locations. Eighty-three percent of these patients were evaluated using a standardized assessment for language. Examining specific adherence to the administration of the Full WAB for L MCA/ACA CVA patients, however, demonstrated 33% adherence, with the remaining 50% receiving the WAB bedside. See Table 1 for additional adherence rates.

**Table 1:** Adherence to Spaulding Rehabilitation Assessment Protocol (percentages).

			**Stroke program administration August 2018–December 2018**	**Hospital-wide administration January 2019–August 2019**		
			**Stroke program admissions**	**Stroke program admissions**	**Other rehabilitation program admissions**	TOTAL hospital admissions^*^
**L MCA/ACA CVA**
	**Admission**
		**Adherence to protocol**				
		Language measure (WAB-R)	33%	31%	2%	27%
		Cognitive-linguistic measure (CLQT)	68%	61%	35%	52%
		**Rate of administration of alternative measures**				
		Language measure (WAB-bedside)	50%	40%	31%	37%
	**Re-evaluation**
		**Adherence to protocol**				
		Language measure (WAB-bedside)	38%	22%	5%	16%
		Cognitive-linguistic measure (CLQT)	25%	14%	35%	52%
		**Rate of administration of alternative measures**				
		Language measure (WAB-R)	9%	8%	2%	6%
	**Admission**
**Other CVA Location**	
		**Adherence to protocol**				
		Language measure (WAB-bedside)	51%	53%	28%	43%
		Cognitive-linguistic measures (CLQT)	63%	67%	31%	52%
		**Rate of administration of alternative measures**				
		Language measure (WAB-R)	1%	2%	0%	1%
	**Re-evaluation**
		**Adherence to protocol**				
		Language measure (WAB-bedside)	20%	8%	0%	4%
		Cognitive-linguistic measure (CLQT)	28%	13%	5%	9%
		**Rate of administration of alternative measures**				
		Language measure (WAB-R)	2%	1%	0%	<1%

* *TOTAL Hospital Admissions includes Stroke and Rehabilitation Program Admissions*.

For Implementation 3.0 Hospital (January 2019–August 2019) there were a total of 402 stroke admissions hospital-wide with 170 and 232 admissions for L MCA/ACA strokes and other stroke locations, respectively. Sixty-four percent of these patients were evaluated using a standardized assessment for language. Examining specific adherence to the administration of the Full WAB for L MCA/ACA CVA patients, however, demonstrated 27% adherence, with the remaining 37% receiving the WAB bedside.

### Part III—Pilot Evaluation of Data Collected Through Standardized Assessments to Begin to Evaluate Predictive Models of Language Recovery After Stroke

One of the long-term goals of the collaborative standardization of evaluations is to contribute to a language database that can be used to inform recovery predictions of language in the acute phase of recovery. In order to begin to evaluate data, we conducted pilot analyses over cases with at least two time points of evaluation.

## Methods

### Participants

Records from standardized language evaluations completed at BWH and SRH per clinical protocol were retrieved for patients who were evaluated at a minimum of two timepoints between June 2017 and July 2019. To be included in pilot analyses, patients had to be native English speakers, 18 years of age or older, and have sustained a left MCA stroke that could have extended into anterior cerebral artery (ACA) and posterior cerebral artery (PCA) territory within the same hemisphere. Patients with prior history of stroke or comorbidities including developmental delay or other significant neurologic history (e.g., neurodegenerative disorder) were excluded. See Table 2 for demographic and stroke-related information, including WAB Aphasia Quotient (AQ) and Aphasia Classification information across timepoints.

**Table 2:** Demographic and stroke-related information for eligible cases.

				**BWH evaluation**				**SRH ADMISSION evaluation**				**SRH re-evaluation**			
**Case #**	**Age range**	**NIHSS**	**Lesion location**	**Days: stroke to eval**	**WAB type**	**WAB AQ**	**WAB aphasia classification**	**Days: stroke to eval**	**WAB type**	**WAB AQ**	**WAB aphasia classification**	**Days: stroke to eval**	**WAB type**	**WAB AQ**	**WAB aphasia classification**
1	70–74	1	Posterior	4	B	85	Anomic	7	F	98.4	No Aphasia				
2	85–89	29	Both	1	B	80	Anomic	5	F	94.6	Anomic				
3	90–94	20	Posterior	3	B	40.8	Conduction	6	F	55.5	Conduction				
4	55–59	4	Both	1	B	38.3	Broca's	8	F	79.4	TCM				
5	65–69	15	Both	2	B	36.7	Broca's	3	F	37.5	Broca's				
6	80–84	4	Both	1	B	31.7	Wernicke's	6	F	45.2	Wernicke's				
7	80–84	4	Both	0	B	24.2	Wernicke's	5	B	^**^	Fluent				
8	40–44	6	Both	1	B	20	Broca's	5	B	^**^	Broca's				
9	80–84	11	Both	3	B	19.2	Broca's	4	F	11.9	Broca's				
10	70–74	5	Anterior	2	B	55.8	TCM	5	F	78	Anomic	14	B	90.8	Anomic
11	80–84	19	Both	4	B	48.3	Broca's	10	F	68.5	TCM	37	B	96.7	No Aphasia
12	80–84	–	Posterior	1	B	41.7	Wernicke's	4	B	40	Wernicke's	23	B	26.7	Wernicke's
13	65–69	–	Both	14	B	39.2	Broca's	16	F	34.4	Broca's	37	F	43.8	Broca's
14	85–89	16	Anterior	6	B	19.2	Broca's	14	F	18.6	Broca's	33	F	^**^	Broca's
15	70–74	22	Both	14	B	10	Global	16	F	7.2	Broca's	30	B	15	Broca's
16	55–59	29	Both	7	B	^*^	Global	16	F	11.3	Global	29	B	34.2	Broca's
17	75–79	8	Anterior					5	F	80.8	Anomic	15	B	91.7	Anomic
18	50–54	13	*L BG*					6	F	79.5	TCM	17	F	94.3	Anomic
19	50–54	18	Anterior					4	B	78.3	Anomic	22	B	97.5	Anomic
20	45–49	17	*L IVH*					19	B	75.8	TCS	34	B	76.7	Conduction
21	50–54	7	Anterior					14	B	67.5	Anomic	31	B	85	Anomic
22	70–74	3	Anterior					14	B	61.7	Anomic	27	B	80.8	Anomic
23	85–89	7	*L MCA*					12	F	61.2	Broca's	28	B	86.7	Anomic
24	65–69	9	*Posterior*					11	F	59.1	TCS	34	B	91.7	Anomic
25	65–69	–	*L MCA*					22	F	58.9	TCM	41	F	77.6	Anomic
26	80–84	–	Both					6	F	56.6	Wernicke's	18	F	70.3	TCS
27	40–44	10	Anterior					20	F	48.9	TCM	28	B	53.3	Broca's
28	60–64	6	*L MCA/PCA*					34	F	32.4	Wernicke's	57	F	35.1	Wernicke's
29	75–79	2	Anterior					5	F	27.2	Broca's	17	B	57.5	Broca's
30	25–29	22	*L MCA/PCA*					17	B	25	Broca's	35	B	53.3	Broca's
31	80–84	8	Both					8	B	23.3	Wernicke's	20	B	24.2	Wernicke's
32	55–59	22	Both					8	F	22	Global	20	F	35.1	TC-Mixed
33	55–59	18	*Both*					16	F	12.1	Global	50	F	18	Global
34	90–94	–	*L MCA*					10	F	9.7	Broca's	24	B	20	Broca's
35	45–49	–	*L MCA*					9	F	7.5	Global	19	B	32.5	Broca's
36	75–79	18	*L MCA*					5	F	0	Global	25	F	34.7	Global
37	40–44	25	*L MCA*					17	F	^**^	Global	30	B	38.3	Broca's

*Age range rather than specific age is reported and sex is omitted from this table for confidentiality purposes. Lesion Location information is reported based on the radiology report from the medical record; in cases where the radiology report was not available, location was obtained from the clinical note and is reported in italics—this information is included for information purposes only and is not included in statistical analyses. Severity ratings for Aphasia Quotient (AQ) are as follows: 0–25 Severe-Profound; 26–50 Severe, 51–75 Moderate; 76+ Mild. “B” denotes WAB Bedside version; “F” denotes WAB Full version*.

* *Denotes participant was unable to pass screener to yield AQ*;

** *Denotes missing subdomain scores, impacting calculation of AQ*.

Of the 796 database entries, 37 patients met inclusion criteria and were evaluated at two or more timepoints. Of these 37 patients, 9 were evaluated at BWH admission, then again at SRH admission, while another 7 were evaluated at all three timepoints: BWH admission, SRH admission, and SRH re-evaluation. The remaining 21 patients received evaluation at the two SRH timepoints, SRH admission and SRH re-evaluation. In addition to language evaluation data, patient age, sex, NIH Stroke Scale NIHSS) score, receipt of Tissue Plasminogen activator (tPA), date of stroke, and date of hospital admission were retrieved from the database.

### Radiology Scan Information

Radiology reports and clinical scans (MRI) were retrieved from the Partners HealthCare Research Patient Data Registry (RPDR) for all patients whose acute care hospitalization was within the Partners HealthCare Network. Clinical scans were retrieved with the intent of completing lesion masking (outlining the lesion) and calculating lesion volume and location based on regions of interest. The fact that these were clinical scans, however, presented several challenges for lesion masking and normalization. Motion artifacts were present in many samples and structural scans varied in their alignment, slice resolution, and whole-brain coverage, with many of the higher resolution scans only including partial brains. It was determined that reliable lesion volumes would not be obtainable from these non-standardized scans, therefore based on the lesion information outlined in radiology reports, as well as clinical scan data, lesions were classified at anterior lesions, posterior lesions, or both anterior/posterior lesions. Classifications were reviewed by two study staff. For patients admitted to SRH from a hospital outside the Partners HealthCare Network for whom radiology reports were not available, lesion data was retrieved from clinical notes within the medical record for informational purposes only and this lesion data was not included in statistical analyses, with the exception of two patients for whom complete radiology report information was available.

### Data Analysis

Statistical analyses were preformed using R Software for Statistical Computing (37). The first set of analyses examined the dependent variable, SRH Admission AQ. Data on this dependent measure were available from 34 patients, as three of the patients in our sample were missing a WAB subdomain score, impacting calculation of an AQ. Regression analyses were run in a forward selection manner to evaluate the relationship between independent and dependent variables, and strength of potential models, entering up to three variables due to our sample size. Variables were entered into the model based on their hypothesized predictability as reported in the literature and on correlation strength with the dependent variable. The first regression evaluated aphasia severity (AQ) accounting for days post-onset of evaluation. Then, additional models were evaluated in a step-up manner, adding lesion location, coded as anterior/posterior only or both, and NIHSS. We then ran a second set of analyses using a different outcome variable: aphasia severity (AQ) at SRH re-evaluation.

In addition to pilot regression analyses, we were interested in examining the proportion of maximal recovery made by each patient. Given that patients varied in their initial severity, a proportional maximal recovery was computed for each patient to account for the differences in potential change. This was calculated as the observed change, or difference between scores, divided by the maximum potential change (T2 severity – T1 severity)/(severity score maximum - T1 severity) (28, 38). An important limitation to address here is a lack of consistency over whether the WAB-Bedside or Full WAB was entered into this comparison. Both tests yield an Aphasia Quotient and according to the WAB Testing manual, interpretation of the WAB-Bedside sections and tasks are consistent with the full test (35), suggesting that a comparison is possible, but should be interpreted with caution.

## Results

Correlation across continuous variables of interest was assessed (Table 3). A very strong negative correlation was observed between time (number of days from stroke until rehabilitation admission evaluation) and aphasia severity (AQ) at all three timepoints. A strong correlation was observed between time and NIHSS, and a minimal to moderate correlation was observed between time and age. NIHSS was moderate-strongly negatively correlated with initial BWH acute care severity, however, the correlation was observed to be less strong by the time of SRH re-evaluation. Predictor variables were not highly correlated with each other.

**Table 3:** Correlation matrix between continuous variables of interest.

	**Age**	**NIHSS**	**BWH AQ**	**Days to SRH eval 1**	**SRH eval 1 AQ**	**SRH eval 2 AQ**
Age	1	0.46	−0.27	0.43	−0.27	−0.34
NIHSS		1	−0.69	0.87	−0.66	−0.46
BWH AQ			1	−0.96	1	0.94
Days to SRH Eval 1				1	−0.95	−0.84
SRH Eval 1 AQ					1	0.96
SRH Eval 2 AQ						1

*Shading reflects the relative strength of correlations, with darker shading indicating a stronger correlation*.

### Predictors of SRH Admission AQ and SRH Re-Evaluation AQ

Regression analysis with days post-onset of evaluation as the predictor and SRH Admission AQ as the outcome variable only accounted for 3% of the variance, and was not statistically significant (*p* = 0.321). Consistent with prior studies, when lesion was included in the model as a predictor, the model was statistically significant, accounting for 26.0% of the variance in SRH Admission AQ [*F*_(2, 22)_ =3.871, *p* = 0.03]. NIHSS, which was the next most highly correlated variable was added to the model and contributed to an R-squared change of 5.4%. Though this model accounted for a larger percentage of the variance, the model was not significant [F_(3, 18)_ = 2.75, *p* = 0.07].

Regression analysis with days post-onset of evaluation as the predictor, and SRH Re-Evaluation AQ as the outcome variable, was not statistically significant (*p* = 0.285) and only accounted for 4.4% of the variance in the model. Including lesion in the model as a predictor explained an additional 14.3% of the variance in SRH Re-Evaluation AQ, but was again not statistically significant [*F*_(2, 15)_ = 1.728, *p* = 0.21]. Similarly, the addition of NIHSS explained an additional 9.6% of the variance, but the model was not statistically significant.

### Language Severity Change

**Figure 3:**
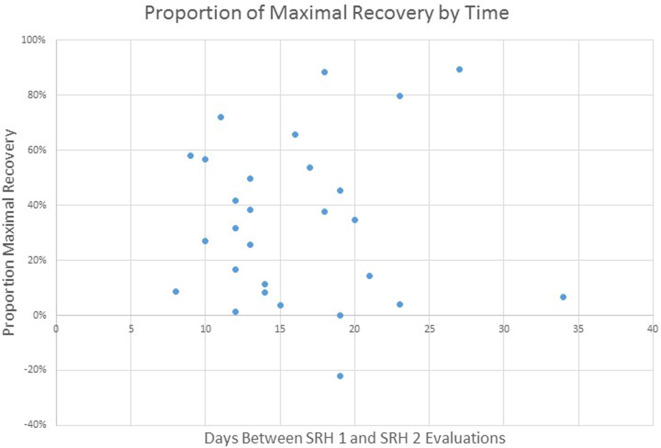
WAB AQ proportion of maximal recovery from SRH initial evaluation to re-evaluation as a function of time (days) between evaluations.

**Figure 4:**
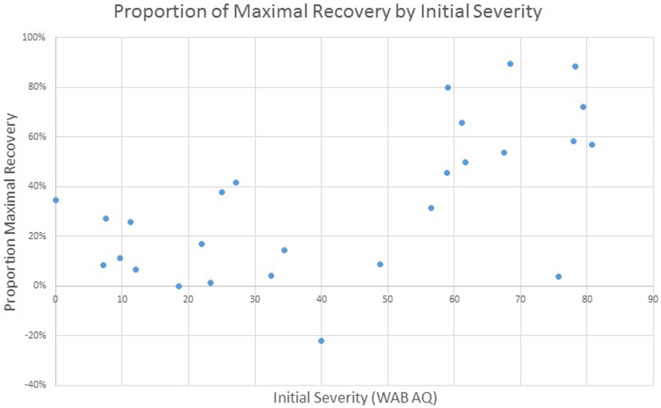
WAB AQ proportion of maximal recovery from SRH initial evaluation to re-evaluation as a function of severity at initial assessment.

Given the focus of this project on the implementation of standardized language assessment measures in acute care and inpatient rehabilitation, we were interested in examining the proportion of maximal recovery made by individual patients. Comparisons of aphasia severity at SRH Admission and SRH re-evaluation showed a wide variety of proportion change ranging from 1% proportion maximal recovery to 89% proportion maximal recovery. The correlation between time between evaluations (as measured in days) and change was not significant, *r*_(27)_ = 0.01, *p* = 0.95 (see Figure 3). The correlation of proportion maximum recovery and aphasia severity at initial evaluation was significant *r*_(27)_ = 0.62, *p* <0.001 (see Figure 4). Individuals with lower aphasia severity scores corresponding to more severe language impairment showed more limited proportion recovery over this limited timeframe.

## Discussion

Results from the current study demonstrate that implementing standardized processes for the evaluation of language is feasible in acute care and inpatient rehabilitation settings, though an ideal process has yet to be identified, particularly for the inpatient rehabilitation setting. For the acute care setting, adherence rates to a standardized protocol over a 2-year period demonstrated 70% adherence. In a survey regarding practice patterns, 70.1% of clinicians reported completing informal assessment measures and 51.1% reported using individualized assessments developed by clinicians or the institution (1) in the acute stages post-stroke, thus 70% adherence over a two-year period with limited reinforcement measures is encouraging. Follow-up conversations with clinicians have revealed that in some cases, computers were not available in rooms, sessions were interrupted by other caregivers or exclusionary conditions, such as evaluating non-English speaking patients prevented the complete administration of the bedside WAB. Clinicians continue to express satisfaction with the measure, stating that the administration is efficient and informative and that using a standardized vocabulary across caregivers is helpful for patient hand-off. In this acute practice setting, the primary needs are to determine the presence or absence of aphasia, administer a diagnosis, initiate therapy and determine the next level of recommended care, conditions satisfied by the measure. Importantly, clinicians continue to supplement the standardized protocol, evaluating additional cognitive-linguistic domains based on their clinical judgment.

Within the inpatient rehabilitation setting, the QIF framework, and collaborative approach, has led to multiple iterations of implementation. Overall results of this initiative demonstrated that administering a standardized assessment in inpatient rehabilitation is feasible, with standardized language or cognitive assessment being completed upon admission for between 52 and 71% of patients in the Stroke Program. Clinician adherence is consistent with rates reported in studies that examine standardized assessment practices within other rehabilitation disciplines, such as physical therapy [48–66% adherence (39, 40)], and those specifically examining post-stroke standardized assessment practice patterns [52–88% adherence (41, 42)]. Incorporating measurable outcomes into clinical practice has been recognized as important for evaluating the effect of interventions, quality of care, advancing knowledge and policy (43–45). While standardization initiatives represented changes in practice, changes were feasible and adhered to over time in acute care.

The iterative process of implementation, however, revealed challenges identifying a suitable language measure for all patients. Initially, the WAB-Bedside was judged to be too abbreviated for L MCA CVA patients in the inpatient rehabilitation setting, yet closer examination once the Full WAB was recommended revealed low rates of administration. This indicates a need to revisit assessment procedures to improve adherence in a way that supports clinical data collection and decision-making. The inpatient rehabilitation setting offers more time for evaluation relative to acute care, but these evaluations establish foundations for goals targeted over a longer period of time than in acute care and that must ready the patient, in many cases, for discharge home. Language interventions are often characterized as being either impairment-based, focusing on stimulating impaired subdomains of speaking, listening, reading, or writing; or communication-based, focused on building functional communication through a variety of methods (46). The WAB is an impairment-based measure, which may not capture the range of deficits and abilities important to evaluate when selecting a combination of impairment-based and communication-based interventions, particularly for patients returning home or to work and resuming activities of daily life [e.g., work demands, finances, group and/or social activities, routine home activities see (47)]. While clinicians expressed an interested in utilizing the full WAB, it may be that on a case-by-base basis the more abbreviated bedside WAB, which provides an overall evaluation of language ability, accompanied by more comprehensive impairment-based testing of specific domains and/or evaluations of communication functioning was better suited than the full WAB. In the acute rehabilitation setting, language evaluations are used to plan interventions that must stimulate the language system and also provide access to functional communication sufficient for the home, work or next level of care. The inclusion of functional measures should be considered in future iterations of implementation as they may more appropriately capture patient performance and level of functioning, important for guiding planning for participation at the next level of care.

Interestingly, clinician adherence to the standardized protocol was higher for the CLQT than for language assessment in both the stroke program and the hospital. This may reflect the fact that there are fewer alternate assessments of cognitive abilities that are suitable for stroke and individuals with language deficits. This may also reflect the importance of insights gained from the assessment of cognitive domains on intervention goals at this level of care. Clinicians are tasked with making initial recommendations regarding discharge planning early in each patient's rehabilitation stay. Discharge recommendations (e.g., discharge home independently, 24-h supervision, or skilled nursing services) go beyond considerations of language ability to consider level of cognitive functioning and safety, making cognitive evaluations meaningful.

Based on the data obtained through standardized assessment of language skills across settings, initial model evaluations over pilot data support previous studies that have found that lesion location and size are predictive of outcomes (31, 33, 48–50). Though limited in power, models that incorporated lesion location accounted for the largest degree of variance. Initial evaluation of proportion maximal recovery demonstrated greater proportion of recovery for individuals with lower severities of aphasia at initial assessment, consistent with prior studies which have shown that patients with more severe levels of impairment show more limited improvement (28, 51).

The current evaluation of predictors of outcomes was only preliminary given the small sample size. Furthermore, the assessment measures incorporated in the current implementation were impairment-based measures that present potential limitations. We propose that an improved understanding of the predictors of recovery will come through consideration of both impairment-based and functional outcome measures. Next steps in evaluating appropriate outcome measures should also examine practice patterns to better understand how outcome measures are utilized to guide intervention planning, as information obtained in assessments needs to be deemed meaningful to clinical practice. Clinical-decision making tools, such as algorithms have been shown to reduce variability in clinical care practices and improve patient outcomes (52). Guidelines that help align outcome measurement with treatment selection, however, are not readily available to guide aphasia assessment and intervention practices.

Additionally, future work will involve exploring metrics obtained by other disciplines, including physical and occupational therapy, through interdisciplinary partnerships to identify what measures are meaningful and clinically-feasible. Adoption into routine clinical practice offers the potential to contribute data that can then be evaluated via new predictive models of improvement. While analyses of data collected in a clinical context may not advance knowledge in the same manner as highly-controlled empirical studies, enlisting clinicians, and creating practice-based evidence may inform the research trajectory and contribute pilot data or systematic observations that can lead to the development of well-controlled empirical studies, which can then feed back into clinical practice. A pattern of practice, evaluation, analysis, and knowledge transfer has the potential to result in research findings that more readily translate into clinical practice, strengthening the bridge that links research and practice.

## Data Availability Statement

The datasets generated for this study are available on request to the corresponding author.

## Ethics Statement

The studies involving human participants were reviewed and approved by Partners IRB. Written informed consent for participation was not required for this study in accordance with the national legislation and the institutional requirements.

## Author Contributions

MS, SV-R, and CT-H organized the database. MS performed statistical analyses and wrote the first draft of the manuscript. SV-R and CT-H wrote sections of the manuscript. All authors contributed to the conception and design of the study and contributed to the manuscript review and revision.

## Conflict of Interest

The authors declare that the research was conducted in the absence of any commercial or financial relationships that could be construed as a potential conflict of interest.
