# Supplementary material for: Implementing a Standardized Language Evaluation in the Acute Phases of Aphasia: Linking Evidence-Based Practice and Practice-Based Evidence
Source: Front Neurol. 2020 Jun 1;11:412. doi: 10.3389/fneur.2020.00412 (PMC7278284; doi:10.3389/fneur.2020.00412)
Supplement: Supplementary file 1 [file Data_Sheet_1.pdf]

**Detailed query details for Partners RPDR billing data requests**

Query # 1:

- Encounter detail\Hospital of service: Brigham and Women's Hospital
- From 07/1/2017 to 8/31/2019
- And
    - 18 – 34 years old
    - 35 – 44 years old
    - 45 – 54 years old
    - Or
    - 55 – 64 years old
    - 65 to 74 years old
    - 75 to 84 years old
    - >= 85 years old
- And
    - **Speech and language deficits following cerebral infarction:** ICD10:I69.32
    - **Speech and language deficits following nontraumatic intracerebral hemorrhage:** ICD10:I69.12
    - **Speech and language deficits following nontraumatic subarachnoid hemorrhage:** ICD10:I69.02
    - **Speech and language deficits following other cerebrovascular disease:** ICD10:I69.82
    - **Speech and language deficits following other nontraumatic intracranial hemorrhage:** ICD10:I69.22
    - Or
    - **Speech and language deficits following unspecified cerebrovascular disease:** ICD10:I69.92
    - **Other speech and language deficits following cerebral infarction:** ICD10:I69.328
    - **Other speech and language deficits following nontraumatic subarachnoid hemorrhage:** ICD10:I69.028
    - **Other speech and language deficits following other cerebrovascular disease:** ICD10:I69.828
    - **Other speech and language deficits following other nontraumatic intracranial hemorrhage :** ICD10:I69.228
    - **Other speech and language deficits following unspecified cerebrovascular disease:** ICD10:I69.928
    - **Aphasia:** ICD10:R47.01
    - **Aphasia following cerebral infarction:** ICD10:I69.320
    - **Aphasia following nontraumatic intracerebral hemorrhage**: ICD10:I69.120
    - **Aphasia following nontraumatic subarachnoid hemorrhage:** ICD10:I69.020
    - **Aphasia following other cerebrovascular disease:** ICD10:I69.820
    - **Aphasia following other nontraumatic intracranial hemorrhage:** ICD10:I69.220
    - **Aphasia following unspecified cerebrovascular disease:** ICD10:I69.920
    - **Primary progressive aphasia:** ICD10:G31.01

Total number of cases returned: **2189**

Query # 2:

- Encounter detail\Hospital of service: Brigham and Women's Hospital
- From 07/1/2017 to 8/31/2019
- And
  - 18 – 34 years old
  - 35 – 44 years old
  - 45 – 54 years old
  - 55 – 64 years old
  - 65 to 74 years old
  - 75 to 84 years old
  - >= 85 years old

Or

- And
  - **Age-related cognitive decline:** ICD10:R41.81
  - **Cognitive communication deficit:** ICD10:R41.841
  - **Cognitive deficits following cerebral infarction:** ICD10:I69.31
  - **Cognitive deficits following nontraumatic intracerebral hemorrhage:** ICD10:I69.11
  - **Cognitive deficits following nontraumatic subarachnoid hemorrhage:** ICD10:I69.01
  - **Cognitive deficits following other cerebrovascular disease:** ICD10:I69.81
  - **Cognitive deficits following other nontraumatic intracranial hemorrhage:** ICD10:I69.21
  - **Cognitive deficits following unspecified cerebrovascular disease:** ICD10:I69.91
  - **Cognitive social or emotional deficit following cerebral infarction:** ICD10:I69.315
  - **Mild cognitive impairment, so stated:** ICD10:G31.84
  - **Other specified cognitive deficit:** ICD10:R41.84
  - **Other symptoms and signs involving cognitive functions and awareness:** ICD10:R41.89
  - **Other symptoms and signs involving cognitive functions and awareness:** ICD10:R41
  - **Other symptoms and signs involving cognitive functions and awareness:** ICD10:R41.8
  - **Other symptoms and signs involving cognitive functions following cerebral infarction:** ICD10:I69.318
  - **Unspecified symptoms and signs involving cognitive functions and awareness:** ICD10:R41.9
  - **Unspecified symptoms and signs involving cognitive functions following cerebral infarction:** ICD10:I69.319
  - **Unspecified symptoms and signs involving cognitive functions following nontraumatic intracerebral hemorrhage:** ICD10:I69.119
  - **Unspecified symptoms and signs involving cognitive functions following unspecified cerebrovascular disease: ICD10:I69.919**

Or

Total number of cases returned: **19375**

Query # 3:

- Encounter detail\Hospital of service: Brigham and Women's Hospital
- From 07/1/2017 to 8/31/2019
- And

  Or
  - 18 – 34 years old
  - 35 – 44 years old
  - 45 – 54 years old
  - 55 – 64 years old
  - 65 to 74 years old
  - 75 to 84 years old
  - >= 85 years old
- And

  Or
  - **Benign neoplasm of brain and other parts of central nervous system :** ICD10:D33
  - **Benign neoplasm of meninges :** ICD10:D32
  - **Malignant neoplasm of brain:** ICD10:C71
  - **Malignant neoplasm of meninges:** ICD10:C70
  - **Malignant neoplasm of spinal cord, cranial nerves and other parts of central nervous system:** ICD10:C72

Total number of cases returned: **5683**
