# Supplementary material for: Implementing a Standardized Language Evaluation in the Acute Phases of Aphasia: Linking Evidence-Based Practice and Practice-Based Evidence
Source: Front Neurol. 2020 Jun 1;11:412. doi: 10.3389/fneur.2020.00412 (PMC7278284; doi:10.3389/fneur.2020.00412)
Supplement: Supplementary file 2 [file Data_Sheet_2.pdf]

Stroke Outcomes Measure Survey

April 2018 Feedback: Please complete these questions to help us better understand how we use the updated Stroke Outcomes Measure. Our goal of the survey is two-fold--we're interested in 1) the implementation of the updated measure and how it's impacting our workflow and 2) how we can improve the measure itself. Thanks for your time!

CURRENT USE OF THE STROKE OUTCOMES MEASURE

1. **How many language assessments do you perform per month?**
Mark only one oval.

- ( ) 0
- ( ) 1-2
- ( ) 3-5
- ( ) >5

2. **I currently use the Stroke Outcomes Measure for all of my language evaluations.**
Mark only one oval.

	1	2	3	4	5	
Strongly disagree	( )	( )	( )	( )	( )	Strongly agree

3. **How confident are you in your ability to complete a language assessment in the rehab setting?**
Mark only one oval.

	1	2	3	4	5	
Not confident	( )	( )	( )	( )	( )	Very confident

4. **Of the 60 minutes you're in the room completing a speech-language-cognitive evaluation, how many minutes are you spending on the standardized Stroke Outcomes Measure?**
Mark only one oval.

- ( ) 1-10 minutes
- ( ) 11-20 minutes
- ( ) 21-30 minutes
- ( ) 31-40 minutes
- ( ) 41-50 minutes
- ( ) 51-60 minutes

5. **How long does a typical language assessment report take to write (i.e. time spent documenting in Epic and writing report)?**
Mark only one oval.

- 20-30 minutes
- 31-40 minutes
- 41-50 minutes
- 51-60 minutes
- 60+ minutes

6. **I feel that my report clearly reflects the data I collected during the evaluation.**
Mark only one oval.

	1	2	3	4	5	
Strongly disagree						Strongly agree

7. **I feel that my report paints a clear picture of the patient's current level of function.**
Mark only one oval.

	1	2	3	4	5	
Strongly disagree						Strongly agree

8. **I feel that my report can be easily interpreted by future clinicians to assess a patient's progress.**
Mark only one oval.

	1	2	3	4	5	
Strongly disagree						Strongly agree

9. **After an evaluation, I am confident diagnosing aphasia subtype (e.g. aphasia as Broca’s, Wernicke’s, Transcortical Motor, Conduction, etc)**
Mark only one oval.

	1	2	3	4	5	
Strongly disagree						Strongly agree

10. **My confidence diagnosing aphasia subtype has improved since adopting the Stroke Outcomes Measure.**
Mark only one oval.

	1	2	3	4	5	
Strongly disagree						Strongly agree

EVALUATING THE MEASURE

11. **I believe that obtaining standardized measures on all stroke patients is important.**
Mark only one oval.

	1	2	3	4	5	
Strongly disagree	⬭	⬭	⬭	⬭	⬭	Strongly agree

12. **I feel that the Stroke Outcomes Measure provides meaningful information towards my characterization of patients**
Mark only one oval.

	1	2	3	4	5	
Strongly disagree	⬭	⬭	⬭	⬭	⬭	Strongly agree

13. **I feel that the Stroke Outcomes Measures is an efficient use of time with my patients.**
Mark only one oval.

	1	2	3	4	5	
Strongly disagree	⬭	⬭	⬭	⬭	⬭	Strongly agree

14. **The Stroke Outcomes Measure contributes to ___ % of my overall assessment of the patient's language and cognitive abilities (e.g. Does the measure represent 80% of your assessment because you present additional tests or 100% of your assessment?)**
Mark only one oval.

- ⬭ 0-15%
- ⬭ 16-30%
- ⬭ 31-45%
- ⬭ 46-60%
- ⬭ 61-75%
- ⬭ 76-90%
- ⬭ 91-100%
- ⬭ Other: ______________________

15. **What other assessments/subtests do you typically use in conjunction with the Stroke Outcomes Measure?**

____
____
____
____
____

16. **What's working? Please indicate what you find useful in the measure.**

17. **What still needs improvement? Please indicate what barriers you encounter and ways the measure could be improved. Are there items that aren't helpful? Are there items you'd like to add?**

Powered by
Google Forms
