# Supplementary material for: Implementing a Standardized Language Evaluation in the Acute Phases of Aphasia: Linking Evidence-Based Practice and Practice-Based Evidence
Source: Front Neurol. 2020 Jun 1;11:412. doi: 10.3389/fneur.2020.00412 (PMC7278284; doi:10.3389/fneur.2020.00412)
Supplement: Supplementary file 3 [file Image_1.pdf]

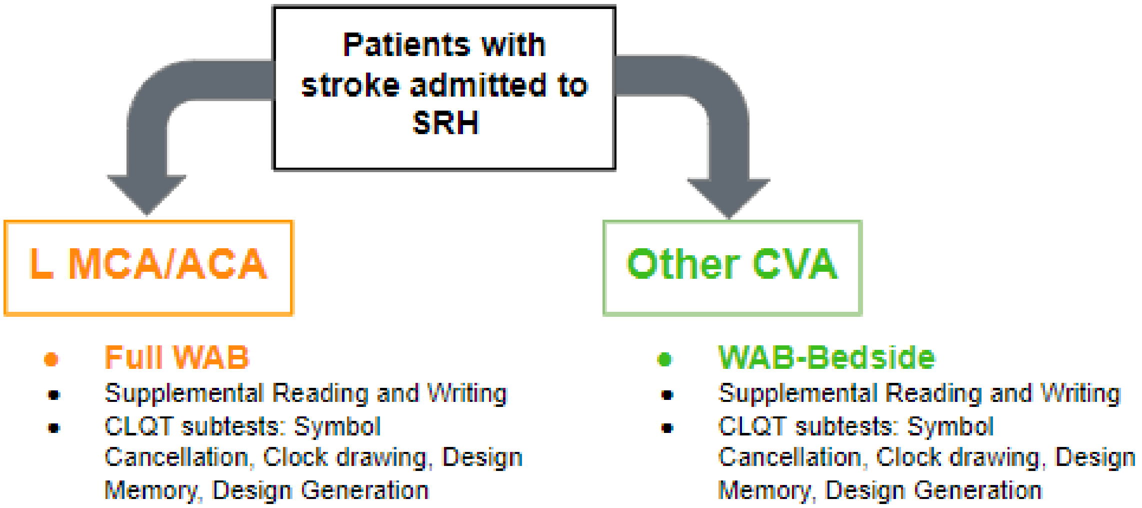

Supplemental Figure 1: Assessment pathway for patients admitted with a stroke to Spaulding Rehabilitation Hospital
